# Pathological sulcus vocalis: treatment approaches and voice outcomes in 36 patients

**DOI:** 10.1007/s00405-018-5040-2

**Published:** 2018-08-28

**Authors:** Beata Miaśkiewicz, Agata Szkiełkowska, Elżbieta Gos, Aleksandra Panasiewicz, Elżbieta Włodarczyk, Piotr H. Skarżyński

**Affiliations:** 1 Otorhinolaryngology Surgery Clinic, Institute of Physiology and Pathology of Hearing, Kajetany, Warsaw, Poland; 2 Audiology and Phoniatrics Clinic, Institute of Physiology and Pathology of Hearing, Kajetany, Warsaw, Poland; 3 Audiology and Phoniatrics Faculty, Fryderyk Chopin University of Music, Warsaw, Poland; 4 World Hearing Center, Institute of Physiology and Pathology of Hearing, Kajetany, Warsaw, Poland; 5 Heart Failure and Cardiac Rehabilitation Department, Second Faculty, Medical University of Warsaw, Warsaw, Poland; 6 Institute of Sensory Organs, Kajetany, Warsaw, Poland

**Keywords:** Sulcus vocalis, Sulcus glottidis, Microlaryngoscopy, Injection laryngoplasty, Hyaluronic acid

## Abstract

**Purpose:**

This is a retrospective study to evaluate the results of surgical treatment of patients with pathological sulcus vocalis.

**Methods:**

Thirty-six patients with pathological sulcus underwent surgery and in 33 cases were performed additional injection laryngoplasty. The pre- and postoperative evaluation of patients included the GRBAS scale, stroboscopic, and objective acoustic voice assessment. The Voice Handicap Index questionnaire (VHI-30) was also used and the scores were obtained from 33 patients.

**Results:**

The stroboscopic evaluation showed significant improvement of amplitude, mucosal wave, and glottal closure after treatment (*p* < 0.001). The VHI-30 scores decreased considerably indicating improvement due to the treatment for all aspects measured by VHI (*p* < 0.05, or *p* < 0.01). In all domains of GRBAS scale, the differences between preoperative and postoperative assessment were statistically significant (*p* < 0.001). We observed a significant change in Shim and APQ parameters (*p* < 0.05). Improvement was also observed in the sAPQ parameter, but it was not statistically significant (*p* = 0.051). For the remaining acoustic parameters, no changes were observed.

**Conclusions:**

The surgical procedure with supplementary injection laryngoplasty of the vocal folds is a good treatment option for pathological sulcus vocalis. The post-treatment self-assessment indicates the significant improvement in VHI, just as perceptual–acoustic evaluation of voice does. Patients with pathological sulcus frequently present with amplitude disturbances, what explains their significant improvement after treatment.

## Introduction

Sulcus vocalis is a laryngeal condition linked to a clinically inhomogeneous defect of the covering epithelium with structural malformation of the vocal fold, ranging from minor invagination to the deep focal pits.

The classifications used today were introduced by Bouchayer and Cornut [1], and Ford [2]. The two main types of pathological sulci are vergeture (type 2) and open cyst (type 3). Vergeture refers to an atrophic groove under the free edge of the vocal fold; sulcus 3 manifests as a pocket lined with a thick epithelium which goes as deep as the vocal ligament or muscle [1]. Ford and colleagues [2] extended this classification to account for the variability in clinical appearance and distinguished the physiologic sulcus (type 1) with normal or minimally altered mucosal wave and intact layered structure of the lamina propria.

There is a very wide range of incidence of sulcus vocalis ranging from 0.4 to 48% [3–6].

The etiology of sulcus vocalis is still controversial. Bouchayer and colleagues speculated that the origin of sulcus was congenital and a result of the fourth and sixth branchial arch anomalies [1]. There have also been reports of familial occurrence [7]. Nakayama and colleagues found high incidence (48%) of sulcus deformities in pathological examinations for laryngeal cancer, and suggested an acquired origin resulted from local trauma and/or chronic inflammation [8]. A mechanism similar to the development of middle ear cholesteatoma was considered by Lee et al. [9]. The heterogeneity in origin and clinical appearance makes the diagnostics and treatment of sulci a challenge [10–12]. There are many treatment modalities for sulcus, but all of them aim to improve voice quality by diminishing the glottal gap and restoring mucosal wave propagation and the symmetry of vibration [1, 2, 12, 13].

The main goal of this study was to evaluate the results of surgical treatment of patients with pathological sulcus.

## Materials and methods

**Fig. 1:**
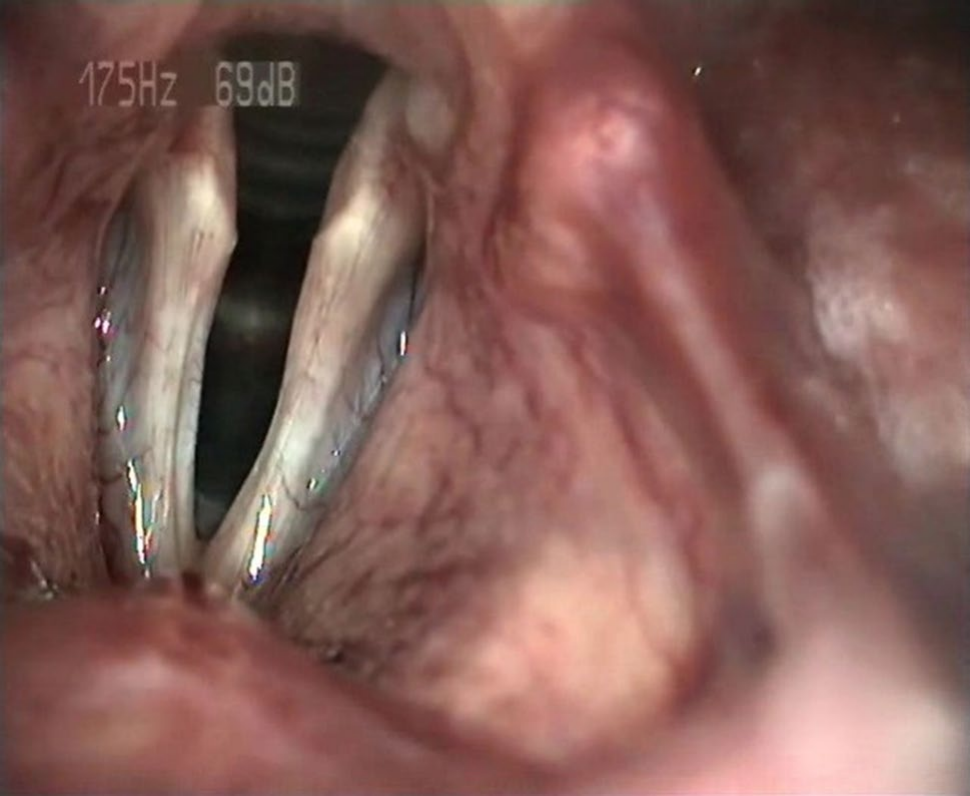
Bilateral sulcus type 2 (LVS)

**Fig. 2:**
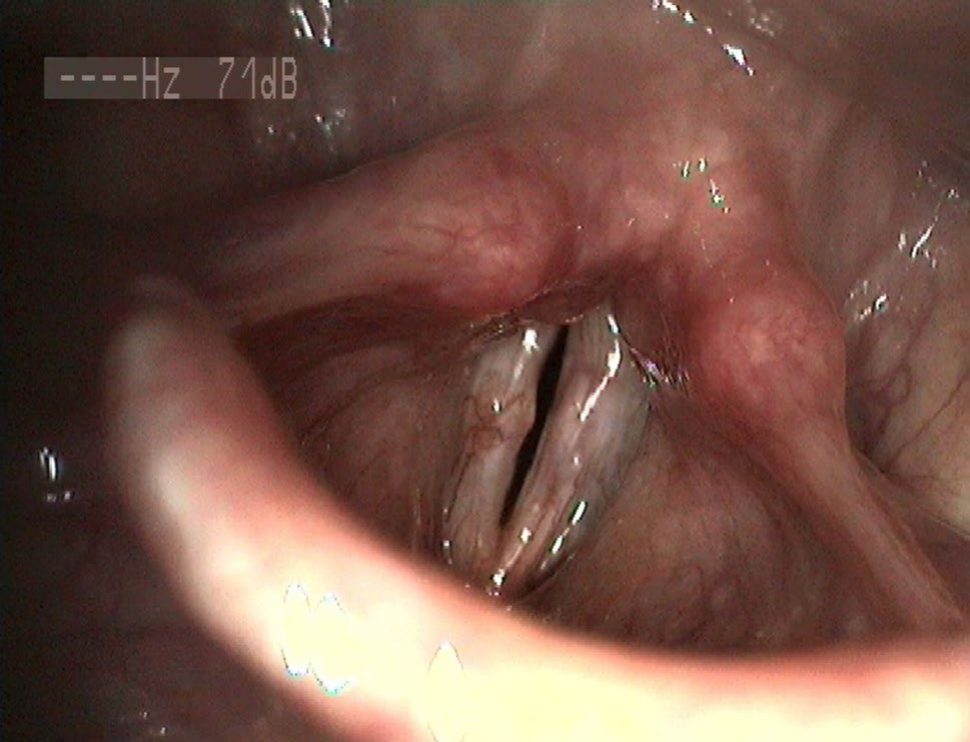
Bilateral sulcus type 3 (LVS)

Thirty-six patients with diagnosis of pathological sulcus, treated surgically between 2011 and 2016, were enrolled in this study. The diagnosis was made by a laryngologist–phoniatrist following laryngovideostroboscopic examination and confirmed or revised during microlaryngoscopy. Based on the final diagnosis, there were 22 subjects with type 2 sulcus (Fig. 1) and 14 with type 3 (Fig. 2). Twelve patients were diagnosed with unilateral sulcus, and 24 patients with bilateral sulci. The group consisted of 23 women and 13 men aged from 22 to 70 years (*M* = 44.17; SD = 11.95). Women were aged from 32 to 70 years (*M* = 43.91; SD = 10.88), and men were aged from 22 to 67 years (*M* = 44.62; SD = 14.12).

Patients reported hoarseness, diminished voice intensity and range of voice, vocal fatigue, and strained, breathy, or unstable voice.

The preoperative patient evaluation included psychosocial, auditory-perceptual, acoustic, and laryngovideostroboscopic (LVS) assessment. All diagnostic tests in 36 patients were repeated at 8–12 months postoperatively, but only 7 patients had the follow-up period longer than 1 year after surgery; thus, the group was too small to do the long-term follow-up analysis.

The Voice Handicap Index questionnaire (VHI-30) was used [14] and the scores were obtained from 33 patients. VHI total score, and emotional, physical, and functional subscale scores were calculated.

An auditory-perceptual evaluation of patients’ voices was carried out with the use of the GRBAS scale [15] which estimates the grade of hoarseness (G), roughness (R), breathiness (B), asthenia (A), and strain in the voice (S) on a scale from 0 to 3 (0, normal; 1, mild; 2, moderate; 3, severe). Ratings, based on a patient’s sustained phonation and a short speech sample, were made by three experienced phoniatrists and the score averaged.

The objective acoustic voice analysis was performed with a Computerized Speech Lab (CSL) 4500 external module from KAY Elemetrics Corporation (Lincoln Park NJ). All voices were recorded with an ECM 800 microphone (Behringer) positioned approximately 15 cm from the mouth, at an angle of 45°, to reduce airflow effects. Analysis of a voice sample recorded at a sample rate of 25 kHz was done using Multidimensional Voice Program software (MDVP 5105 version 2.7.0). Three samples of the sustained vowel “a” in modal voice were used for analysis; only the middle portion of the uttered vowel was used (min. 0,6 s), avoiding onset and offset effects [16–20]. The following acoustic parameters were calculated: average fundamental frequency (F0), frequency variations (% Jitter;  Relative Average Perturbation, RAP;  Pitch Perturbation Quotient, PPQ;  Smoothed Pitch Perturbation Quotient, sPPQ;  Fundamental Frequency Coefficient Variation, vFo), amplitude variations (% Shimmer;  Amplitude Perturbation Quotient, APQ;  Smoothed Amplitude Perturbation Quotient, sAPQ;  Peak-to-Peak Amplitude Coefficient of Variation, vAm), and noise-related parameters (Noise-to-Harmonic Ratio, NHR; Soft Phonation  Index, SPI).

The pre- and postoperative LVS tests were performed by the operating surgeon—laryngologist—phoniatrist (BM) with a 70° rigid laryngoscope (EndoStrob DX Xion 327, GmBH, Germany), and glottal closure and vibration characteristics of the vocal folds were assessed subjectively. Each studied stroboscopic pattern was evaluated on a scale from 0 to 3; for glottal closure (0, complete; 1, small gap; 2, moderate gap; 3, large gap); amplitude (0, normal; 1, mildly diminished; 2, moderately diminished; 3, severely diminished); and mucosal wave (0, normal; 1, mildly restricted; 2, moderately restricted; 3, completely lacking).

Operations were performed under general anesthesia with suspended microlaryngoscopy and endotracheal intubation. The vocal folds were inspected under magnification with an operating microscope and palpated with a blunt instrument to assess the sulcus morphology. The surgical technique was based on a concept by Bouchayer and Cornut with Remacle’s modification [1, 21, 22]. The incision and resection of the tissue were done with an Acublade CO2 laser (Lumenis, Santa Clara, CA) using superpulse mode. As concerns the working parameters of the laser the length of the straight line was 1–2 mm, a penetration depth of 0.2 mm with using a double scan mode. The power was calculated by software and was set around 10 W. The dissection or undermining of the vocal fold soft tissues was performed with cold instruments.

For sulcus type 2, the free edge of the vocal fold was grasped with a Bouchayer microforceps (Micro-France, France) and pulled medially to enhance the vergeture, and an epithelial incision was made along the superior margin of the sulcus. Grasping the edge of the vergeture, then blunt dissection was proceeded in the superficial plane to the ligament and inferomedial edge of the sulcus to free the epithelial attachments. During this procedure, we were aware of respecting and preserving the epithelium. Occasionally, in cases of epithelium tearing, we were forced to excise the remained atrophic tissue.

**Fig. 3:**
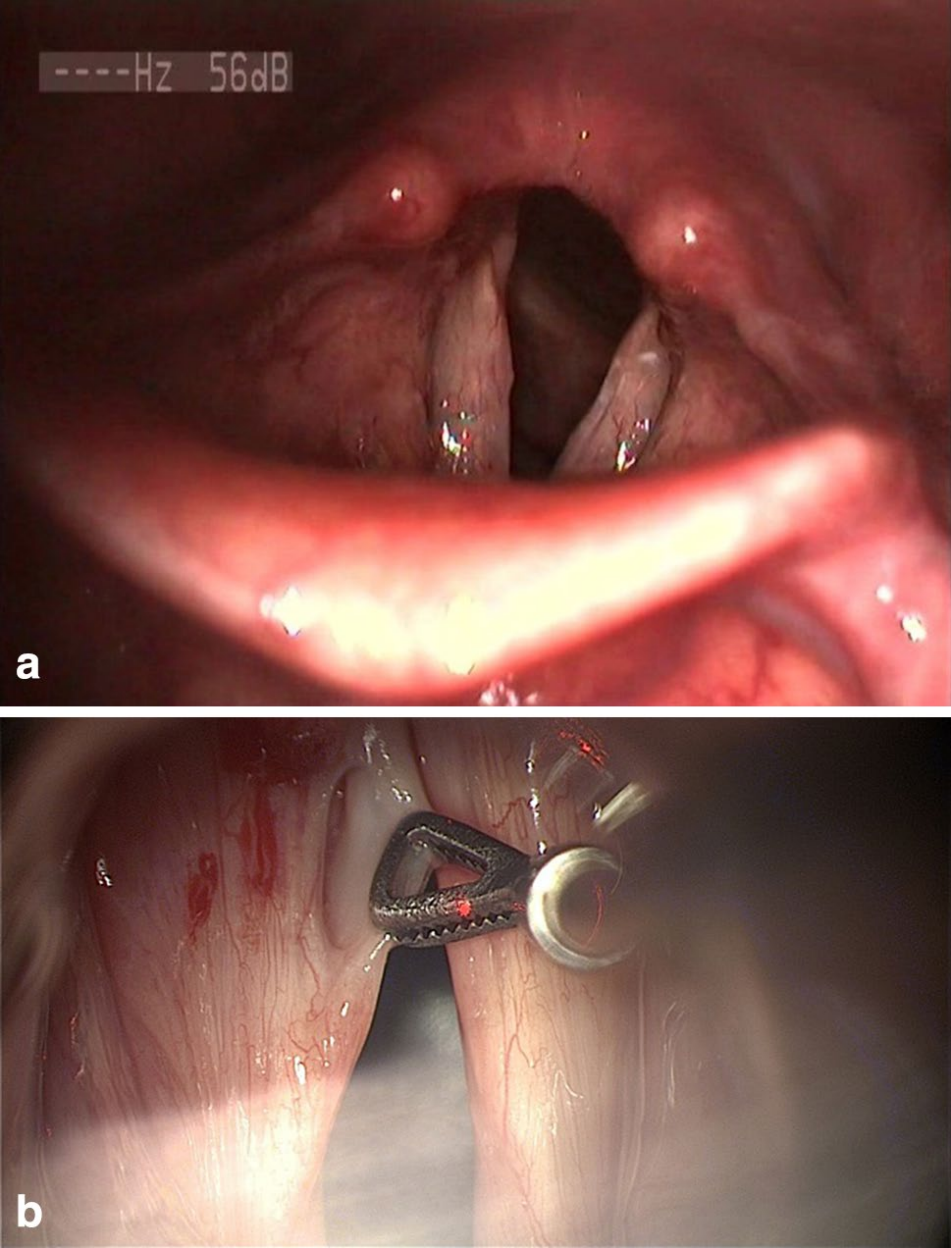
**a** Sulcus type 3 of the left vocal fold (LVS). **b** Same patient (intraoperative view)

For sulcus type 3 (Fig. 3a, b), the lower margin of the sulcus was grasped with microforceps and an epithelial incision started from the superior lip closely around the pocket opening. Dissection proceeded deeply until the ligament was reached to separate the walls and the fundus from surrounding tissue, and then, the entire sulcus pocket was excised. Saline solution and adrenalin were applied with cottonoid for tissue cooling and hemostasis.

In all cases, we applied fibrin glue (Tissucol, Baxter, Vienna) to facilitate approximation of the incised epithelial edges, protect deeper planes, and improve healing process.

If there was a significant vocal fold atrophy, injection laryngoplasty was performed during the same operation. Independently of the type of sulcus, the main criterion of supplementary vocal fold augmentation was the width of the glottal gap assessed subjectively by the operating surgeon during LVS performed the day before operation. The additional criterion was atrophic appearance of the vocal fold after sulcus removal. We used two injectable materials: hyaluronic acid (HA, Surgiderm 24 XP, Allergan) and calcium hydroxylapatite (CaHa, Radiesse Voice Implant, Merck). We applied either one of two substances alone or combination of them. The areas of injection were different for each material in view of different properties of each substance. HA was injected as close as possible to the deep layer of the lamina propria, until the volume of the vocal fold was assessed as close to normal. The points of injection were adjusted to the configuration of the glottal gap, usually 2 points. The quantity of HA ranged between 0.2 and 0.7 ml (mean 0.45 ml). The most common injection approach in the case of CaHa involved application lateral to the superior arcuate line at the posterior third and/or mid-membranous vocal fold in 1–2 points. The amount of injectable material depended on individual indications and was evaluated as sufficient when the edge of the vocal fold met the midline, [22] and ranged between 0.3 and 1.0 ml (mean 0.77 ml). When bilateral lesion was present, both vocal folds underwent the procedure during the same operation.

Prior to surgery 52% of patients underwent speech therapy without satisfactory voice improvement. Other 48% of patients could not attend the preoperative therapeutic sessions due to a distance from a place of living, lack of time or they were referred to surgery with other than sulcus diagnosis. All patients were informed about a long postoperative recovery period. Postsurgical voice therapy was mandatory in all subjects and involved one session a week for 2–5 months or patients were referred to hospitalization with voice rehabilitation.

### Statistical analysis

Statistical analysis was performed to compare the level of investigated variables before and after the treatment. The results for GRBAS and for assessment of glottal closure, amplitude and mucosal wave are categorical variables, so for comparison of the preoperative and postoperative scores, the test of marginal homogeneity was used for *m* × *n* tables (*m, n* > 2). The VHI scores and acoustic parameters are quantitative variables, so to compare preoperative and postoperative results, the *t* test for paired samples or the Wilcoxon signed rank test was applied. First, the assumption of normality for quantitative variables was checked using the Kolmogorov–Smirnov test. If this assumption was met, a parametric *t* test for paired samples was used. When the compared variables did not fit a normal distribution, a Wilcoxon signed rank test was applied. Test results were reported as significant for *p* < 0.05. The IBM SPSS software version 24 was used for all statistical analyses.

## Results

Three patients with sulcus type 3 underwent isolated surgical procedure without additional augmentation. In other 33 cases, we performed supplementary medialization laryngoplasty during the same surgical stage. In 29 patients, we applied HA as the only injectable material; in 2 subjects, CaHa only and in 2 individuals a combination of HA and CaHa.

All patients were instructed to rest their voice for 5–7 days after surgery. Antibiotics (amoxicyllin with clavulanic acid) were prescribed for 5 days and PPI for 2–4 weeks. All patients reported poor voice for 1–2 months postoperatively, but they were warned beforehand that this was likely.

Data on pre- and postoperative LVS patterns are presented in Table 1. There are number of the patients with a specific state of glottal closure, amplitude, and mucosal wave before surgery in the rows. In addition, in the columns, we can see how many of them changed their status in terms of assessment of LVS patterns after surgery.

**Table 1:** Comparison of preoperative and postoperative assessment of LVS patterns

Glottal closure preoperative	Complete	Small gap	Moderate gap	Large gap	
Complete	1	0	0	0	MH = 5.15; *p* < *0.001*
Small gap	15	7	0	0	
Moderate gap	2	11	0	0	
Large gap	0	0	0	0	

Amplitude preoperative	Normal	Mildly diminished	Moderately diminished	Severely diminished	
Normal	2	0	0	0	MH = 5.09; *p* < *0.001*
Mildly diminished	8	5	0	0	
Moderately diminished	4	13	0	0	
Severely diminished	0	3	1	0	

Mucosal wave preoperative	Normal	Mildly restricted	Moderately restricted	Severely restricted	
Normal	0	0	0	0	MH = 5.47; *p* < *0.001*
Mildly restricted	1	3	0	0	
Moderately restricted	0	23	1	0	
Severely restricted	0	3	5	0	

*MH* standardized statistic of marginal homogeneity test; *p-value* observed significance level

The condition of glottal gap was significantly better after the surgery. In addition, amplitude and mucosal wave values were significantly improved postoperatively.

The results of statistical analysis showed that the VHI scores decreased considerably indicating improvement due to the treatment for all aspects measured by VHI (functional, emotional and physical; see Table 2). In addition, the total VHI score decreased significantly from a preoperative value of 44.36 ± 22.76 to a postoperative value of 34.12 ± 24.55. All differences were statistically significant (*p* < 0.05 or *p* < 0.01).

**Table 2:** Comparison of preoperative and postoperative VHI scores

	Preoperative		Postoperative		Test statistic	*p* value
	*M*	SD	*M*	SD		
Functional	11.48	7.58	9.21	7.59	*Z* = 2.15	*0.031*
Emotional	13.52	9.88	9.85	8.81	*Z* = 2.64	*0.008*
Physical	19.36	8.52	15.03	9.34	*t* = 2.87	*0.007*
VHI total	44.36	22.76	34.12	24.55	*t* = 3.16	*0.003*

*M* mean, *SD* standard deviation, *t* result of *t* test; *Z* result of Wilcoxon test; *p-value* observed significance level

Table 3 presents the data concerning pre- and postoperative assessment using GRBAS scale. There are a number of the patients with a specific state of GRBAS parameters before surgery in the rows. In the columns, we can see how many of them changed their status in terms of assessment of GRBAS parameters after surgery.

**Table 3:** Comparison of preoperative and postoperative GRBAS parameters

	Normal voice	Mild	Moderate	Severe	
Grade preoperative					
Normal voice	0	0	0	0	MH = 4.02; *p* < *0.001*
Mild	1	12	1	0	
Moderate	0	19	2	0	
Severe	1	0	1	0	
Roughness preoperative					
Normal voice	1	0	0	0	MH = 4.39; *p* < *0.001*
Mild	3	6	1	0	
Moderate	6	11	4	0	
Severe	1	3	0	0	
Breathiness preoperative					
Normal voice	9	0	0	0	MH = 3.80; *p* < *0.001*
Mild	12	11	0	0	
Moderate	3	1	0	0	
Severe	0	0	0	0	
Asthenia preoperative					
Normal voice	14	0	0	0	MH = 4.47; *p* < *0.001*
Mild	20	2	0	0	
Moderate	0	0	0	0	
Severe	0	0	0	0	
Strain preoperative					
Normal voice	4	0	0	0	MH = 4.56; *p* < *0.001*
Mild	17	7	0	0	
Moderate	2	2	2	0	
Severe	0	2	0	0	

*MH* standardized statistic of marginal homogeneity test; *p-value* observed significance level

In all domains of GRBAS, the differences between preoperative and postoperative assessment were statistically significant (*p* < 0.001). Subjective evaluation of grade, roughness, breathiness, asthenia, and strain of voice improved considerably postoperatively.

Table 4 presents the results of comparison of the values of the voice parameters during the pre- and postoperative periods.

**Table 4:** Comparison of preoperative and postoperative MDVP parameters

	Preoperative		Postoperative		Test statistic	*p* value
	*M*	SD	*M*	SD		
F0	200.20	49.25	209.05	43.16	*t* = 1.37	*0.179*
Jitt	1.83	1.17	1.63	1.00	*Z* = 1.41	*0.159*
RAP	1.09	0.69	0.97	0.60	*Z* = 1.38	*0.169*
PPQ	1.10	0.72	0.94	0.58	*Z* = 1.49	*0.136*
sPPQ	1.39	0.76	1.53	2.35	*Z* = 1.47	*0.140*
vF0	3.17	2.28	3.32	3.94	*Z* = 0.79	*0.432*
Shim	6.12	2.91	5.26	2.48	*Z* = 2.23	*0.026*
APQ	4.54	2.32	3.71	1.59	*Z* = 2.64	*0.008*
sAPQ	6.97	4.42	5.86	3.51	*Z* = 1.95	*0.051*
vAm	20.10	9.44	19.97	11.71	*Z* = 0.70	*0.481*
NHR	0.16	0.06	0.15	0.05	*Z* = 1.44	*0.149*
SPI	13.86	5.73	13.61	5.17	*t* = 0.41	*0.682*

*M* mean, *SD* standard deviation, *t* result of *t* test, *Z* result of Wilcoxon test; *p-value* observed significance level

The analysis showed that there was a significant change in Shim and APQ parameters. They improved significantly after surgery (*p* < 0.05). Improvement was also observed in the sAPQ parameter, but it was not statistically significant (*p* = 0.051). For the remaining parameters no changes were observed.

### Additional analysis

In view of the discrepancies between the results of acoustic measurements reported by other authors, we decided to closely examine this issue [11, 23–25]. We assumed an improvement would not be expected in all patients, but only in those who had a considerably high value of a given parameter before surgery. On the other hand, in patients, where a given parameter was not increased preoperatively, we would not expect to see a change after the operation. We employed normative thresholds of the acoustic parameters as proposed by Delijsky [26] as well as norms provided by KAY Elemetrics Corporation which refer to the adults in general [27, 28]. For each parameter, we separately determined a cut-off point, on the basis of which we separated the patients into two groups: one with low preoperative values of given parameter (lower or equal to the normative value) and another with high values (above the norm).

Table 5 presents the results of a comparison of the pre- and postoperative levels of the MDVP parameters in patients with high values of them before surgery. Wilcoxon’s test was used for analysis.

**Table 5:** Comparison of preoperative and postoperative MDVP parameters in patients with a high value of the listed parameter before the operation

Parameter		Preoperative level		Postoperative level		Test statistic	*p* value
		*M*	SD	*M*	SD		
Jitt	High (*n* = 23)	2.43	1.06	1.59	0.75	*Z* = 3.22	*0.001*
RAP	High (*n* = 22)	1.48	0.60	0.98	0.48	*Z* = 3.07	*0.002*
PPQ	High (*n* = 18)	1.65	0.64	0.96	0.34	*t* = 3.90	*0.001*
sPPQ	High (*n* = 21)	1.81	0.74	1.31	0.72	*Z* = 2.66	*0.008*
vF0	High (*n* = 33)	3.37	2.28	3.49	4.07	*Z* = 1.05	*0.296*
Shim	High (*n* = 31)	6.60	2.84	5.36	2.21	*Z* = 2.47	*0.014*
APQ	High (*n* = 27)	5.17	2.36	3.81	1.54	*Z* = 3.26	*0.001*
sAPQ	High (*n* = 29)	7.74	4.61	6.11	3.84	*Z* = 2.55	*0.011*
vAm	High (*n* = 35)	20.45	9.35	20.12	11.84	*Z* = 0.86	*0.388*
NHR	High (*n* = 4)	0.30	0.06	0.13	0.03	*Z* = 1.83	*0.068*
SPI	High (*n* = 18)	18.80	2.84	15.55	5.28	*t* = 2.57	*0.020*

*M* mean, *SD* standard deviation, *t* result of *t* test; *Z* result of Wilcoxon test; *p-value* observed significance level

In patients with high preoperative values, we saw a significant change in assumed direction, i.e., the values of the parameter decreased considerably after treatment. In most of the parameters, we observed a statistically significant improvement after surgery (*p* < 0.05). In addition, an improvement was found in the NHR parameter, but it was not statistically significant (*p* = 0.068). In the case of vF0 and vAm, no changes were observed.

## Discussion

The spectrum of disease in sulcus cases is diverse. The more advanced the sulcus, the more the microarchitecture of the vocal fold is disrupted. The aim of most surgical techniques is to remove diseased tissue and better separate the covering epithelium from the vocal ligament, releasing the contracture. Basic guidelines have been described by Bouchayer and Cornut, and have been modified by others [1, 2, 21, 22]. All these methods can be combined with endoscopic or external medialization techniques [24, 25, 29]. Satisfying results have also been reported with the use of the “slicing” technique [30].

In our experience, therapy needs to be tailored to each patient, type of sulcus, and a single treatment modality will not work in all cases. We performed distinct operations for the two types of sulcus, aiming to preserve epithelium in type 2 and excise it in type 3. Most of the patients had supplementary injection laryngoplasty and the decision was made on the basis of the width of the glottal gap during phonation in LVS, and the atrophic appearance of the vocal folds intraoperatively. The choice of the injectable material  firstly was dependent on its availability. Initially, we used only HA, so most of the patients (93.9%) received injection of hyaluronan [11, 31, 32]. Site of injection was determined by properties of the materials. Data from the literature indicate that the level of HA in the extracellular matrix of the lamina propria may decrease in sulcus. Its intracordal injection might promote ingrowth of new collagen, own HA, and fibroblasts, correcting the volume of the vocal folds [33, 34]. Some authors suggest the location of injections may be also important in determining the effect and longevity of HA [32, 35, 36]. They reported long-term voice improvement after hyaluronic acid augmentation in glottal insufficiency [32, 36]. Different from HA, lateral injection of CaHa provided adequate medialization of the vocal fold. Regarding distinct properties of HA and CaHa, we used a combination of them in two patients, and injected CaHa to medialize the vocal fold, and HA to correct the volume. The amount of each substance was assessed intraoperatively and considered as sufficient when the free edge of the vocal fold approached the midline. However, only longer follow-up (over 2–3 years) could really define which surgical technique—medialization or dissection—contributes more to postoperative voice effects.

Postsurgical stroboscopic measurements showed considerable improvement (although no normalization) in vibration amplitude, mucosal wave propagation, and glottal closure, consistent with other reports [22, 23, 25, 29, 37]. This reflected an enhancement of vocal fold pliability.

Deterioration of voice in sulcus cases varies markedly, but the majority of patients had a mild-to-moderate grade of hoarseness, roughness, breathiness, or strained voice [38, 39]. Our results, as with other studies [11, 23, 25, 37], showed a significant improvement of GRBAS scale parameters, although the patients’ voices remained hoarse after treatment.

Our treatment led to significant improvement in just a few objective acoustic parameters-Shim, APQ, and sAPQ. On the other hand, there are only a few papers available in the literature which present acoustic measurements after sulcus treatment, and the postsurgical results are diversified [22–25, 36, 37]. Some authors suggest that glottal incompetence creates an air leak, which affects vibratory amplitude [20]. Amplitude variations are considered the most significant factor determining the severity of the phonation disorder [20]. Such a factor could explain the significant improvement in amplitude parameters in our group, especially since most of our patients had high preoperative values. In work on sulcus vocalis by Hirano, he noticed that the value of PPQ was within the normal range in the majority of cases, whereas the APQ and normalized noise energy values were greater than the limit for a normal population [38]. Our analysis of acoustic parameters allowed us to separate normal values from high levels of each parameter, and comparison of high value cases before and after surgery showed significant improvement in all but vAm and vFo. The NHR did not decrease significantly, but only four subjects had a high level of this parameter. A review of the literature showed that normative values of voice parameters vary according to how the measurements are made and the number of samples. The normative values provided by Kay Elemetrics Corporation we consider as only approximate, and this is a limitation of our analysis. It would be better to build our own database and use a common procedure to obtain repeatable values [20, 26].

Many authors report a large discrepancy between acoustic measurements and VHI [40–42]. Despite the presence of a mild or moderate postsurgical hoarseness, we achieved a significant decrease in total VHI score and in subscale scores, a finding compatible with other reports [23, 25].

## Conclusions

The surgical procedure with supplementary injection laryngoplasty of the vocal folds is a good treatment option for pathological sulcus vocalis. The width of the glottal gap could be considered as a criterion for augmentation.

The post-treatment self-assessment indicates the significant improvement in total and all three VHI domains, just as perceptual—acoustic evaluation of voice does, despite a mild or moderate postsurgical hoarseness. Amplitude acoustic parameters are the most frequently disturbed among patients with pathological sulcus, what explains their significant improvement after treatment.
